# Progress in Tissue Culture and Genetic Transformation of Oil Palm: An Overview

**DOI:** 10.3390/ijms20215353

**Published:** 2019-10-28

**Authors:** Rajesh Yarra, Longfei Jin, Zhihao Zhao, Hongxing Cao

**Affiliations:** 1 Coconut Research Institute, Chinese Academy of Tropical Agricultural Sciences, Wenchang 571339, China; rajeshyarra@rediffmail.com (R.Y.); jlf_0511@163.com (L.J.); zhaozhihao201608@catas.cn (Z.Z.); 2 Institute of Genetics and Developmental Biology, Chinese Academy of Sciences, Beijing 100101, China

**Keywords:** in vitro propagation, *Agrobacterium*, particle bombardment, CRISPR/Cas9

## Abstract

Oil palm (*Elaeis guineensis*, Jacq.) is a prominent vegetable-oil-yielding crop. Cultivating high-yielding oil palm with improved traits is a pre-requisite to meet the increasing demands of palm oil consumption. However, tissue culture and biotechnological approaches can resolve these concerns. Over the past three decades, significant research has been carried out to develop tissue culture and genetic transformation protocols for oil palm. Somatic embryogenesis is an efficient platform for the micropropagation of oil palm on a large scale. In addition, various genetic transformation techniques, including microprojectile bombardment, *Agrobacterium tumefaciens* mediated, Polyethylene glycol mediated mediated, and DNA microinjection, have been developed by optimizing various parameters for the efficient genetic transformation of oil palm. This review mainly emphasizes the methods established for in vitro propagation and genetic transformation of oil palm. Finally, we propose the application of the genome editing tool CRISPR/Cas9 to improve the various traits in this oil yielding crop.

## 1. Introduction

Oil palm species such as *Elaeis guineensis* Jacq. and *Elaeis oleifera* are widely distributed in the regions of Africa and South and Central America. Oil palm species are perennial tropical trees belonging to the Arecaceae family, and these oil palm trees are the most efficient resource for vegetable oil (palm oil and palm kernel oil) production. The oil palm tree is an unbranched tree with a long stout single stem ending with a crown consisting of 40–50 leaves. Usually, the leaves are of a pinnate type and 4–5 m in length. The oil palm generally yields large, spherical, red fruits in bunches up to 2000 fruits, and the oil is mainly extracted from the pulp, as well as kernels of these fruits. The oil yield content is significantly higher in African oil palm (*E. guineensis*) compared with American oil palm (*E. oleifera*). African oil palm has been superficially adopted for commercial cultivation due to its high oil yielding quality. However, American oil palm is a good candidate for hybridization with African oil palm as it has special characteristics such as bud rot disease resistance, slow height increment, and oil rich in unsaturated fatty acids with significant amounts of carotenes and tocotrienols. The interspecific hybrid developed from African oil palm and American oil palm (*E. oleifera* × *E. guineensis*) is principally grown in the regions of South America [1,2]. Oil palm (*Elaeis guineensis* Jacq.) cultivation has emerged as the world’s major cultivated crops, being grown on more than 16 million hectares [3,4,5]. The demand for palm oil has increased and will increase further in the coming years to target more than 200 million tons of the total global production of fats and oils [6,7,8], making palm oil the most traded vegetable oil [9]. Indeed, most palm oil production takes place in Asian countries, especially Indonesia and Malaysia, boosting their economies. Oil palm is also an efficient storehouse for the production of biofuels [10,11].

Tropical regions are the best places for the growth and productivity of oil palm trees. Oil palm trees require temperatures in the range of 30–32 °C for better growth. The growth of oil palm species is severely affected if the temperature dips below 20 °C, but the native African oil palm species is also affected at temperatures above 40 °C. The oil palm is a cross-pollinated tree with a breeding period of over ten years. Oil palm is usually propagated through seeds. However, propagation through seeds cannot be used commercially in a true to type manner. Moreover, the seed germination rate of oil palm is very poor and may take a longer period of germination under natural conditions. Green et al. 2013 [12] published a paper describing the application of heat pre-treatment for accelerating the germination process of oil palm seeds. More than 90% of oil palm planting material is from hybrid seeds [13]. Due to their long lifespans, traditional breeding programs are not the best methods for breeding oil palm species. Practices of vegetative propagation methods are impossible for oil palm, as it does not produce axillary shoots. To overcome these difficulties, the tissue culture approach is a suitable choice for the large-scale production of oil palm trees. However, due to having a single vegetative shoot meristem, it is impossible to propagate through traditional tissue-culture-based methods like nodal proliferation and shoot meristem culture. Alternatively, somatic embryogenesis offers an efficient and attractive approach for the micropropagation of oil palm. Somatic embryogenesis is the best choice for generating uniform clones with desirable elite and true-to-type oil palm plantlet characteristics [14,15]. Moreover, the produced clones have the ability to produce a higher oil content than the commercial seedlings [14,16]. Recent reports clearly demonstrated that oil palm breeding has achieved significant contributions to the enhancement of oil yield, oil quality, and disease resistance [6,17,18,19]. However, this takes a long time. So, genetic transformation techniques can be used to overcome the common barriers to the genetic improvement of oil palm species for improving the oil content and disease resistance. Furthermore, genome editing via CRISPR/CAS9, Zinc-finger nucleases (ZFNs), and transcription activator-like effector nucleases (TALENs) are promising tools for the genetic improvement of oil palm in the near future.

In this review, our aim is to provide a comprehensive summary of the advances in tissue culture and biotechnological methodologies applied for the genetic improvement of oil palm, an important tropical oil crop.

## 2. Oil palm Propagation Methods

To obtain genetically uniform plantlets, vegetative propagation using suckers or cuttings is the best method adopted in many perennial crops. However, vegetative propagation is not possible for oil palm. Alternatively, tissue culture and other biotechnological interventions can be employed to produce oil palm clones without somaclonal variations.

### In Vitro Propagation of Oil Palm

Micropropagation of oil palm by the tissue culture method under sterile environmental conditions often produces true-to-type clones [20,21]. It has been more than 50 years since the first reported in vitro culture study was carried out in oil palm [22,23,24]. After this period, many researchers published papers describing the in vitro propagation of oil palm.

**Figure 1:**
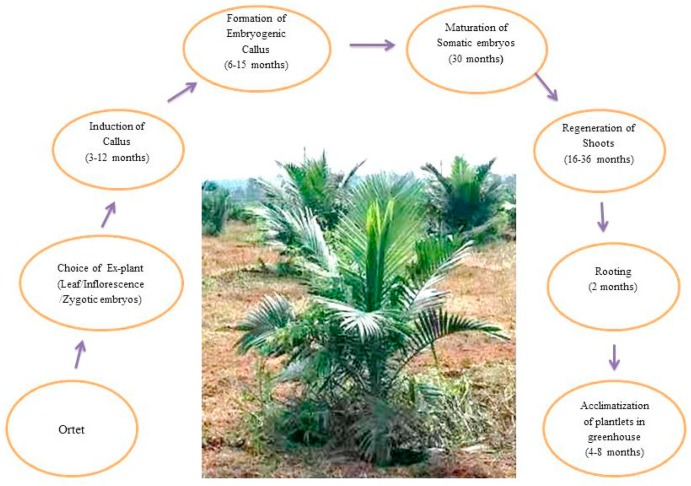
Schematic representation of the phases and time required for each phase in the somatic embryogenesis of oil palm (*Elaeis guineensis*, Jacq.).

Somatic embryogenesis (indirect and direct) is the commonly employed tissue culture approach for the micropropagation of oil palm. Somatic embryogenesis not only safeguards important agronomic features but also maintains the high-grade performance of seedlings. In vitro propagation of oil palm via indirect somatic embryogenesis involves the three main developmental stages: induction of callus from the desired ex-plant, induction of embryoid-like structures (somatic embryos), somatic embryo maturation, and plantlet regeneration (Figure 1). However, it should be kept in mind that the somatic embryogenesis of oil palm is greatly influenced by the type of ex-plant, the composition of media, plant growth regulators, culture conditions, the number and duration of subcultures, and genotype selection [3,10,25,26]. For the induction of embryogenic callus, researchers commonly used two types of plant growth regulators, such as auxins (2,4-dichlorophenoxyacetic acid (2,4-D) and picloram). However, 2,4-D has been extensively used for embryogenic callus (EC) induction [3] (Table 1). Indeed, EC induction is greatly influenced by concentrations of auxins with or without activated charcoal (AC) (9–10 μM without AC or 450 μM with 2.5–3 g/L AC) (Table 1). Furthermore, the embryogenic callus is usually transferred to MS (Murashige and skoog’s) medium supplemented with a decreased auxin (1-naphthaleneacetic acid (NAA)) concentration for the induction and maturation of somatic embryos [3,10,27,28] (Table 1). In addition to NAA, some protocols have described the supplementation of cytokinins in negligible amounts for somatic embryogenesis (SE) induction and as well as for maturation. The occurrence of somaclonal variations is a common indication when cytokinins are included in culture media [3,29]. A few attempts have been made to exclude the somaclonal variations by substituting the cytokinins with polyamines or putrescine or abscisic acid (ABA) [10,30,31] (Table 1). Floral abnormality (mantled flower) is one of the epigenetic changes that occur in micropropagated oil palm plants [31,32,33]. The propagated clones with mantled flowers result in the loss of oil palm productivity.

Micropropagation of oil palm via somatic embryogenesis has been successfully achieved from different explants, such as mature zygotic embryos [25,34,35,36,37] (Table 1), immature zygotic embryos [38,39] (Table 1), immature male inflorescence [10] (Table 1), immature female inflorescence [40,41] (Table 1), mature leaves [20] (Table 1), immature leaves [27,28,31,42,43], young plantlets [10,44,45] (Table 1), and shoots [46] (Table 1). Most of the above-mentioned reports described the propagation of oil palm through indirect somatic embryogenesis, in which the callus is the intervening phase. To date, Jayanthi et al. 2011 [44] have only reported direct embryogenesis in oil palm with an efficiency rate of 80% using young plantlets. The degree of methylation of a specific DNA sequence is directly associated with the embryogenesis rate of ex-plants obtained from ortets. Ho et al. [47] assumed that this degree of methylation might be a powerful biomarker to identify and eliminate recalcitrant ortets regenerated through somatic embryogenesis. Somatic embryogenesis is a valuable and reliable tool for rapid propagation, genetic improvement, and synthetic seed production of oil palm [48].

## 3. Genetic Diversity Conservation of Oil Palm

Plant genetic diversity usually refers to the total number of genetic characteristics in the genetic makeup of a species and is vital for the genetic improvement of crops. It is mandated for the sustainable development of oil palm from the species of ex situ and in situ populations with high genetic diversity [52]. Founding gene or germplasm banks for the conservation and sustainable utilization of plant genetic diversity are a pre-requisite for the genetic improvement of oil palm species. Cryopreservation is one of the best approaches for the conservation of oil palm genetic resources. The cryopreservation method for oil palm genetic resources has been employed for zygotic embryos (ZEs), somatic embryos (SEs), embryogenic cell suspensions, friable embryogenic callus, kernels, seeds, and pollen [53]. Among them, somatic embryos are the best and most popular targets for cryopreservation, as somatic embryogenesis is one of the best-adopted techniques for micropropagation of this important palm species [54]. Genetic diversity conservation of oil palm through the cryopreservation of somatic embryos has been well studied [53]. The developed protocols can be adopted to maintain elite clones of oil palm plants for the preservation of oil palm germplasm for efficient conservation [53,55]. Among the explored genetic diversity conservation methods, the cryopreservation of oil palm somatic embryos can be chosen as an important technique for long-term germplasm conservation. Cryopreservation involves the storage of biologically important samples at low temperatures (−196 °C), usually in liquid nitrogen. This cryopreservation method is the best employed technique to ensure safe and cost-efficient long-term conservation.

## 4. Genetic Transformation of Oil Palm

Genetic transformation is a potent and effective tool for creating transgenic plants, and it has emerged as a remarkable accomplishment in plant biotechnology. Genetic transformation technology has been developed for various crops and has reformed the agricultural sector by enabling the insertion of transgenes into agronomically important species. Transgenic plants exhibit novel traits. Genetic transformation methods are generally mediated by particle bombardment, *Agrobacterium tumefaciens*, and DNA uptake (PEG mediated and microinjection) into protoplasts. Genetic transformation for transgenic plant production mainly relies on the stable integration of foreign DNA into the host genome (stable transformation) and whole plant regeneration from the transformed tissue. However, these two processes are independent but concurrent and are vital for the successful production of transgenic plants. The introduced transgenes are integrated at random sites in the host plant genome.

**Figure 2:**
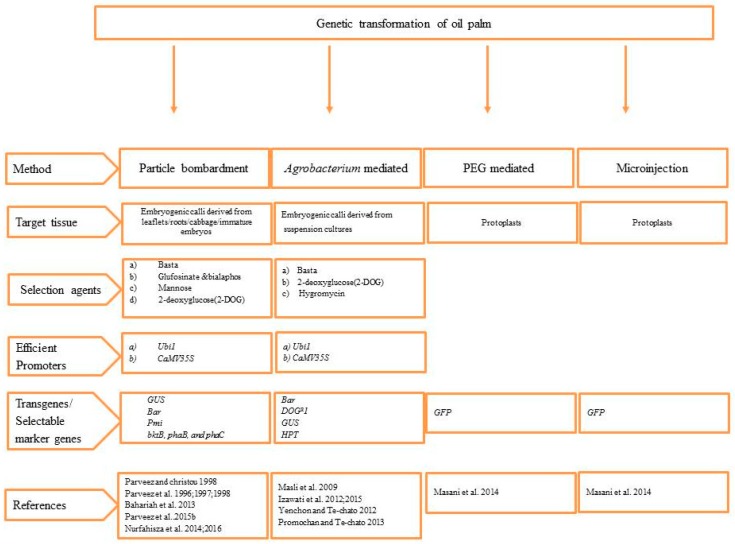
Genetic transformation methods established in oil palm.

For successful genetic transformation, a reliable and efficient regeneration protocol is essential. The two major difficulties concerned with the genetic transformation of oil palm are (1) the method of tissue culture and (2) the selection and regeneration of transgenic plants [56]. Somatic embryogenesis is only an efficient technique for the clonal propagation of oil palm, although the induction and maturation of callus and embryos are very slow and poorly responsive under in vitro conditions [56,57]. Various genetic transformation techniques like particle bombardment, *Agrobacterium*-mediated techniques, and direct uptake of DNA by protoplasts have been developed so far and utilized for the genetic improvement of oil palm (Figure 2). However, particle-bombardment-mediated transformation is the most commonly employed method, as it has been the most successful compared with other methods [6]. The production of transgenic oil palm plants is aimed at increased productivity and improved quality and quantity of palm oil with prospective benefits for the consumers [6,58]. In the past four decades, with improvements in regeneration and transformation methods for oil palm species, extensive research has been carried out to introduce various genes to acquire novel traits for better improvement of oil palm.

### 4.1. Particle Bombardment Mediated Genetic Transformation of Oil Palm

A new method of genetic transformation using high velocity microprojectiles to transfer DNA material into a broad range of plant tissues was developed and first described by John Sanford [59] in epidermal onion cells. The particle bombardment process relies on the direct delivery of DNA coated onto microprojectiles (gold or tungsten) into desired tissue via a gene gun. The first successful stable transformation was developed for tobacco and soybean using the particle bombardment technique [60,61]. The absence of biological incompatibilities is the main advantage of particle-mediated transformation over *Agrobacterium*-mediated transformation. Particle bombardment is not only limited to the transformation of dicots but also explores the genetic transformation of conifers and monocots [62].

#### 4.1.1. Factors Influencing Particle-Bombardment-Mediated Transformation

The success of the particle-bombardment-mediated method of transformation in a specific crop is mainly influenced by different parameters (biological and physical), DNA delivery, selectable marker genes, the availability of suitable promoters, and the use of appropriate selection agents for screening the transgenic plants [6,63,64,65].

#### 4.1.2. Selection of Promoters

In particular, the selection of an appropriate promoter for driving the expression of the transgene in the host genome is most needed. Chowdhury et al. 1997 [65] evaluated five constitutive promoters for the expression of the GUS gene in embryogenic callus and young leaflets of mature plants and seedlings of oil palm using particle-bombardment-mediated transformation. The five constitutive promoters used for evaluation in this study are the recombinant truncated maize alcohol dehydrogenase 1 promoter (*Emu*), the cauliflower mosaic virus 35S promoter (*CaMV 35S*), maize Ubiquitin 1 (*Ubi 1*), maize alcohol dehydrogenase 1 (*Adh 1*), and rice actin 1 (*act 1*). Both histochemical and fluorometric data clearly show that *Ubi 1* and *Emu* are able to drive the expression of transgenes. However, the *Ubi 1* promoter is the most suitable promoter for the stable transformation of oil palm [6,65].

#### 4.1.3. Physical Parameters

The success of the particle-bombardment-mediated transformation of oil palm is also dependent on the physical parameters that affect the efficiency of DNA delivery in different tissues [6,64]. Physical parameters such as the acceleration pressure of helium, the bombardment distance, chamber vacuum pressure, microcarrier size, DNA quantity, number of bombardments, and the effects of spermidine and calcium chloride on microcarrier–DNA binding should be optimized for the efficient transformation of oil palm. Parveez et al. 1997 [64] published a paper on the evaluation of the above-mentioned physical parameters by bombarding the *pEmuGN* construct (GUS gene under the control of *Emu 1* promoter) into an embryogenic callus. These studies clearly show that the DNA delivery efficiency is mainly affected by slight changes in these parameters without altering the vacuum pressure or the number of bombardment shots.

#### 4.1.4. Biological Parameters

The optimization of biological parameters is also being taken into consideration for efficient genetic transformation. Several biological parameters such as explant type, two types of microcarriers (tungsten and gold), time duration between bombardments, bombardment pre-culture, genotype, immature embryo pre-culture, DNA concentration, osmoticum type and concentration, and duration of osmoticum treatment before and after bombardment were evaluated and optimized for particle-mediated transformation of embryogenic calli [6,63]. These parameters were optimized for the transient expression of the GUS gene under the control of the *Emu* promoter [63]. All biological parameters showed significant effects on the transient expression of the GUS gene, except for the genotype parameter. Immature embryos exhibited the highest GUS expression when compared with other explant types used. Even though the authors do not consider immature embryos to be the best targets for particle bombardment, most researchers prefer leaves as explants for the successful initiation of embryogenic calli. The transfer of embryogenic calli to MS medium supplemented with 0.4 M of mannitol for 2 h before bombardment triggered higher transient GUS gene expression than when the control (MS + 0 M mannitol) was used. The transformation efficiency was significant with the bombardments by delivering 1.5 μg of DNA coated on 300 μg of gold microcarriers [63].

#### 4.1.5. Adequate Selection Conditions

Estimating appropriate selective agents to recover genetically transformed cells is vital for oil palm transformation. Therefore, the selection of an effective selective agent is a prerequisite for screening the transformed from the untransformed ones. Selection is typically based on antibiotic or herbicide resistance and the selection agent should not have a deleterious effect on plant growth. Parveez et al. 1996 [66] reported the use of Basta as a selection agent for the genetic transformation of oil palm. This selection agent (Basta) has shown a minimum inhibitory concentration (MIC) of 50mg/L for killing more than 99% of untransformed embryogenic calli [66,67]. To date, researchers have used Basta (50mg/L) as an effective selection agent for oil palm genetic transformation experiments. However, recent reports [68,69] demonstrated the successful use of calli suspension cultures for the production of transgenic oil palms, and this can be recovered on less than 50 mg/L of Basta. Data have shown that concentrations of 10 and 20 mg/L of Basta are required for the growth suppression of embryogenic calli and embryoids (suspension cultures), respectively. Nurfahisza et al. 2016 [69] also published an article on the effective use of other selection agents such as glufosinate-ammonium and bialaphos at 3 mg/L of the MIC for growth suppression of embryogenic calli.

An alternative to the Basta selection system, another selection system (mannose) was developed for the recovery of transgenic oil palm plants [6,70,71]. It was found that 30 g/L of mannose is an optimal dose for the recovery of transgenic plants from the embryogenic calli of oil palm. Baharaiah et al. [71] further demonstrated that the time period required for the recovery of transgenic plants is much less in the mannose selection system when compared with the Basta selection system. These results clearly demonstrate that the mannose is also a promising selection agent for the genetic transformation of oil palm.

Another selection system was also established for oil palm genetic transformation. The selection of transgenic plants is achieved by employing a selection agent, namely, 2-deoxyglucose (2-DOG) [56]. However, this selection system is mainly used for the embryogenic calli derived from the suspension cultures. The ideal concentration optimized for the screening of the putative transformants is 400 mg/L of 2-DOG [6,72].

#### 4.1.6. Production of Oil Palm Transgenics Via Particle-Bombardment-Mediated Transformation

The first stable transformation of oil palm for the production of transgenic plants was reported by Parveez and Christou in 1998 [67]. The embryogenic calli of oil palm plants were bombarded with the plasmid pAHC25 (carrying *gusA* and *bar* genes driven by the ubiquitin promoter), pGH24 (carrying *gusA* and *hpt* genes driven by the CaMV35S promoter), and another plasmid (carrying *gusA*, *hpt* and *bar* genes driven by CaMV35S promoter) using biolistic-mediated transformation. The transgenic plants were recovered on the selection media containing 50 mg/L of Basta. Transgene integration was further verified by PCR and Southern hybridization analyses. The Basta leaf painting assay of the whole regenerated plant also clearly indicated the successful integration of the transgene into the oil palm genome, [67].

Bahariah et al. 2013 [71] published a paper describing the successful production of oil palm transgenics via the mannose selection system. The four transformation vectors (pMI11, pMI11G, pMI3, and pMI3G) carrying the *pmi* gene alone or in combination with the *gusA* gene under the control of either the maize ubiquitin promoter (pMI11 and pMI11G) or the CaMV*35S* promoter (pMI3 and pMI3G) were successfully bombarded into the embryogenic calli to generate transgenic oil palm plants. The transgenic plants were recovered on the selection medium containing mannose (30 g/L). The molecular analysis (PCR, GUS assay, RT-PCR, and Southern blotting) of regenerated plants confirmed the stable integration of transgenes.

Recently, transgenic oil palm plants were generated as a source for the production of biodegradable plastics (PHB, Polyhydroxybutyrate). The plasmid pME22 carrying bacterial PHB biosynthetic genes, namely, *bktB*, *phaB*, and *phaC*, driven by the *Ubi* promoter was transformed into the embryogenic calli of oil palm via particle bombardment [73]. The transformed healthy embryogenic lines were recovered on the herbicide Basta selection system (50 mg/L). Transgene integration and expression were further verified by PCR, Southern blotting, and qRT-PCR experiments. The authors further performed staining analyses (Nile blue A and HPLC) to detect the PHB content in transgenic oil palm. The amount of PHB detected ranged from 0.33 to 0.58 mg/g dwt. The presence of lower PHB amounts in transgenic oil palm led to the normal growth of transgenic compared to control lines [73]. This is a promising consideration for accepting the transgenic oil palm plants in terms of biosafety environmental issues.

### 4.2. Agrobacterium-Mediated Genetic Transformation of Oil Palm

*Agrobacterium*-mediated plant genetic transformation is the gene delivery system in which the bacterium transfers a particular DNA molecule into the host genome [74]. In the early 1980s, the first transgenic dicot plants were successfully produced by *Agrobacterium*-mediated genetic transformation, and these attempts also extended the host range of *Agrobacterium* to monocot members, which are less infected by the *Agrobacterium*. Raineri et al. 1990 [75] published a paper on the production of the first stable monocot transgenic crop (rice) by *Agrobacterium*-mediated transformation. Later, *Agrobacterium*-mediated transformation methods were adopted by various monocotyledonous plants, from cereal crops to ornamental plants [76,77,78]. Similar to monocot plants, in vitro propagation of oil palm via somatic embryogenesis is an efficient approach. However, various factors such as the genotype, explants used for infection, explant pre-culture, tissue wounding before infection, the concentration of acetosyringone during infection, co-cultivation, and different methods of selection influence the efficiency of *Agrobacterium*-mediated transformation.

An attempt has been made to optimize the parameters affecting the *Agrobacterium*-mediated transformation of oil palm via the infection of suspension cultures of embryogenic calli [79]. This study failed to describe the efficiency of transformation. The embryogenic calli were infected with *Agrobacterium* strain LBA4404 harboring the plasmid pUBA consisting of the *bar* gene under the control of the *Ubi 1* promoter. Before *Agrobacterium* infection, the embryogenic calli were pre-treated with medium containing 6% sucrose and 200 µM acetosyringone for an hour to induce virulence genes. Simultaneously, the wounding of embryogenic calli was also attempted by bombarding with microprojectiles to discharge phenolic compounds. The transgenic oil palm plants were screened on the Basta selection system. Molecular analyses (PCR and Southern blotting) of the regenerated transgenic plants showed stable integration of the *bar* gene. Further, a leaf painting assay of transgenic oil palm plants strongly demonstrated the tolerance of transgenic plants of up to 50mg/L of Basta, even after a few years [80].

Izawati et al. [56] published a paper explaining a new selectable marker system, i.e., the 2-deoxyglucose-6-phosphate phosphatase gene (*DOG^R^1*) for *Agrobacterium*-mediated genetic transformation of oil palm. The plasmid pBIDOG containing the *DOG^R^1* gene under the control of the 35S promoter was transformed into embryogenic calli of oil palm via the *A. tumefaciens* strain LBA4404. The transgenic oil palm plants were recovered on the medium containing 400 mg/L of 2-deoxyglucose (2-DOG). Molecular analyses such as PCR and Southern blotting also confirmed the integration of the transgene into the oil palm genome. The transformation efficiency of 1.0% was shown by using this new selection system. The *Agrobacterium*-mediated transformation efficiency of oil palm was similar for both the Basta and 2-DOG selection systems used.

In a previous study [81], the effect of the *Agrobacterium* (AGL-1) density during infection and co-cultivation at different time intervals was examined to improve the transformation efficiency. The *Agrobacterium* strain AGL-1 harboring the transformation vector pCAMBIA1304 carrying *gus* and *hpt* was used to infect the embryogenic callus. The highest transient expression of the *gus* gene (100%) was shown when the embryogenic callus was inoculated for 6 h in *Agrobacterium* suspension at a density of 0.8 (OD600) followed by co-cultivation with 200 µM of acetosyringone in the dark for 3 days and further culturing for 2 weeks on medium containing hygromycin. PCR analysis confirmed the presence of *gus* and *hpt* genes in the embryogenic calli. This study failed to produce stable transformants and was limited to optimizing the *Agrobacterium* density for efficient transformation. Later, another study was conducted to compare the influences of two different strains of *Agrobacterium tumefaciens* (AGL-1 and EHA101) on the transformation efficiency and demonstrated that EHA 101 is the prominent strain [82]. Although many researchers have succeeded in optimizing the various parameters for the *Agrobacterium*-mediated transformation of oil palm, an optimized transformation system for stable transformation is yet to be achieved.

### 4.3. Protoplast Transformation of Oil Palm by PEG-Mediated and Microinjection Techniques

Both particle bombardment and *Agrobacterium*-mediated genetic transformation techniques can be employed to introduce foreign genes into oil palm to achieve stable transformation. However, both techniques not only require optimized physical and biological parameters but also require appropriate selectable markers and promoters for efficient transformation [83]. Apart from these challenges, these transformation methods may promote the integration of transgene copies, either in multiple or low copy numbers. Moreover, being a monocot member, *Agrobacterium*-mediated genetic transformation of oil palm is not efficient compared with particle bombardment. In order to overcome these challenges, protoplasts are the best targets for plant transformation. Protoplasts are plant cells lacking a cell wall that are more permeable to DNA uptake in the transformation process. Besides, many protocols have been established for the isolation of oil palm protoplasts, Masani et al. 2013 [84] described an efficient and reproducible protocol for viable protoplast isolation and regeneration of oil palm plants. The transformation of oil palm protoplasts is a promising and efficient strategy for the production of transgenic oil palm plants. The two types of techniques commonly employed for protoplast transformation are PEG-mediated and micro-injection-based approaches.

Recently, Masani et al. 2014 [85] established the protoplast transformation protocol for oil palm-based PEG-mediated transfection and DNA microinjection. They stated that protoplasts isolated seven days after the subculture of three-month-old embryogenic cell suspension cultures are the best choice for PEG-mediated transformation. Transfection experiments of protoplasts were performed with CFDV-hrGFP plasmid DNA mixed with PEG dissolved in magnesium chloride (MgCl_2_). They achieved a transfection efficiency of 4.22% by validating different parameters such as the heat shock conditions (45 °C/5 min and cooling on ice/1 min) and the concentrations of DNA (50 µg), PEG (25% *w*/*v*), and magnesium chloride (50 mM).

The same research group [85] also reported another oil palm protoplast transformation protocol using DNA microinjection. They adopted the 1% alginate layer for the immobilization of protoplasts for successful transformation. Immobilized protoplasts were incubated in the dark for 3 days at 28 °C and then subjected to microinjection of the linear CFDV-hrGFP fragment. They showed *GFP* expression after 72 h of microinjection. Transfection efficiency of 74.6% was achieved with microinjection of 5 µL of DNA (100 ng/µL concentration). The results clearly indicated that 14% of injected protoplasts developed into the micro calli that were sustained to express *GFP* after 6 months.

## 5. Oil Palm Genome Editing Through CRISPR/Cas9 Technology

Although genetic manipulations via conventional breeding of various crop plants have been utilized to increase crop production and yield, genome editing tools are essential to further increase the crop yield for sustainable food production. Genome editing tools such as TALENs (transcription activator-like effector nucleases), ZFNs (zinc finger nucleases), and (CRISPR)/Cas9 (CRISPR-associated protein 9)-based methods have been effectively applied for targeted genome modifications in various crops [86,87,88]. Genome editing through CRISPR/Cas9 technology is precise, faster, cheaper, and enables highly efficient targeted modification compared with other genome editing tools. Moreover, frequently used transformation methods such as *Agrobacterium*-mediated and particle bombardment techniques can be employed to deliver CRISPR/Cas DNA into the host genome for precise genome modifications. To date, CRISPR/Cas9 technology is applied to improve crop traits such as quality, yield, abiotic and biotic stress resistance. Various conventional breeding and biotechnological approaches have been carried out for the genetic improvement of oil palm, but not much progress has been made. Oil palm productivity is mainly affected by water, temperature, and nutritional stresses [89,90]. For the sustainability of oil palm, the adoption of new technologies is necessary for improving properties such as the oil yield quality, abiotic and biotic stress resistance, and disease resistance. Oil palm genetic improvement through genome editing tools has not been developed so far. However, the genome editing of oil palm through CRISPR/Cas9 is a challenging task because of its huge and complex genome, long breeding cycle, and difficulty with in vitro regeneration and mutant screening.

**Figure 3:**
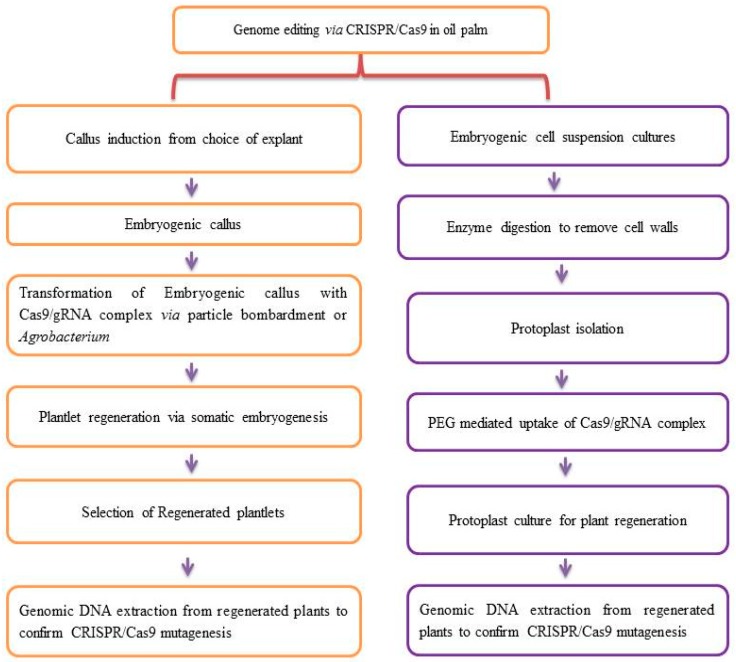
Proposed genome editing methods in oil palm via CRISPR/Cas9.

The successful execution of CRISPR/Cas9 technology in oil palm needs a suitable CRISPR/Cas9 cassette consisting of sgRNA, CRISPR, and efficient Cas9. Genetic transformation methods such as *Agrobacterium*-mediated, particle bombardment, PEG-mediated and microinjection techniques are used as efficient methods for CRISPR/Cas9 applications (Figure 3), and these methods are well established for oil palm genetic transformation. Transforming embryogenic calli with CRISPR/Cas9 DNA may promote bacterial plasmid sequence integration into oil palm, which is against GMO regulations. To address these questions, the employment of CRISPR/Cas9 RNPs approaches may be a better solution for current GMO regulations.

The availability of the oil palm genome sequence [91] paves the way for the improvement of different traits via genome editing by introducing targeted genetic modifications.

## 6. Conclusions

Oil palm is mainly cultivated for vegetable oil production. As oil palm has a long-life cycle, changes in climatic conditions are the main threats for the destruction of the oil palm population. In view of this, it is necessary to develop oil palm cultivars that are resistant to abiotic stresses, diseases, etc., without compromising the yield and quality. The tremendous progress of in vitro propagation techniques has facilitated oil palm clonal propagation as a promising approach for large-scale plant production. Due to the high oil yielding performance, the demand for tissue culture generated plants has certainly increased in recent years. Consequently, somatic embryogenesis is the only efficient method for the clonal multiplication of oil palm. In order to meet the high demand for supply, the adoption of a temporary immersion bioreactor system (R.I.T.A.^®^) is the alternative choice for embryogenic callus multiplication and somatic embryo conversion steps, which are significantly superior to traditional cultivation systems. It is also important to maintain negligible somaclonal variation in micropropagated plants to produce beneficial aspects for growers.

Although genetic transformation techniques are well established for oil palm, more research work is needed for the genetic improvement of oil palm to improve yield and productivity. In recent days, researchers isolated various promoters and useful genes from oil palm. The improvement of the oil yield through genetic engineering is a major challenge to meet the high demand for palm oil. The genome-editing tool CRISPR/Cas9 may play a key role in targeting the genetic modifications of the oil palm genome in the near future. Being sensitive to cold stress, the development of abiotic stress-tolerant transgenic plants is a major breakthrough for the genetic improvement of oil palm. Recent studies [92] have shown that the oil palm *WRKY* genes are highly upregulated under cold stress conditions. Overexpression of these key genes can enhance the tolerance of oil palm to cold stress. Genetic modification of oil palm through genome editing technologies can improve the crop yield and resistance to abiotic stress and various diseases.

## Figures and Tables

**Table 1:** In vitro regeneration of oil palm via somatic embryogenesis.

References	Initial Ex-Plant	Growth Phase	Culture Media & Plant Growth Regulators
Hashim et al. [27]	Young leaf	Callus induction Somatic embryo initiation/proliferation/maturation Plantlet regeneration Rooting	MS + 27–54 µM NAA; 0–4.5 µM 2,4-D MS + Half of the above PGR’s conc. followed by a gradual decrease MS + 0.11 µM NAA MS + 1.1 µM NAA
Gomes et al. [42]	Young leaf	Callus induction Somatic embryogenesis Maturation of SE	MS + 450 µM Picloram + 2.5 g/L Activated Charcoal MS + 12.3 ip; 0.54 NAA MS + 2.5g/L Activated Charcoal
Corrêa et al. [31]	Young leaf	Callus induction Callus Proliferation Somatic embryo initiation/maturation Plantlet regeneration	Y3 + 800 µM 2,4-D + 3.0g/L Activated Charcoal Y3 + 9 µM 2,4-D; 1000 µM Putrescine Y3 + 0.1 µM 2,4-D; 1000 µM Putrescine Y3 + 0.54 µM NAA; 1000 µM Putrescine
Constantin et al. [28]	Young leaf	Callus induction Callus Proliferation Somatic embryo initiation/maturation	MS + 107.41 µM NAA MS + Gradual decrease in NAA
Wan Nur Syuhada et al. [38]	Immature zygotic Embryo	Callus induction Callus Proliferation Somatic embryo initiation/maturation Plantlet regeneration	MS + 9.95 µM 2,4-D MS + 25 µM 2,4-D MS + 0.1 µM NAA MS + 0.1 µM NAA
Thuzar et al. [39]	Immature zygotic Embryo	Callus induction Somatic embryo initiation/maturation Plantlet regeneration	N6 + 9.05 µM 2,4-D N6 + 0.45 µM 2,4-D N6 + 0.5 g/L Activated Charcoal
Monteiro et al. [25]	Mature zygotic Embryo	Callus induction Callus Proliferation Somatic embryo initiation/maturation Plantlet regeneration	MS + 450 µM Picloram + 2.5 g/L Activated Charcoal MS + 5 µM 2,4-D ½ MS + 2.5g/L Activated Charcoal MS
Balzon et al. [37] de Carvalho Silva et al. [36] Gomes et al. [35] Gomes et al. [34]	Mature zygotic Embryo	Callus induction Callus Proliferation Somatic embryo initiation/maturation Plantlet regeneration Rooting	MS + 450 µM Picloram + 2.5g/L Activated Charcoal MS + 40 µM Picloram; 10 µM 2-ip MS + 0.54 µM NAA; 12.3 µM 2-ip + 0–0.3g/L Activated Charcoal ½ MS + 0–2.5g/L Activated Charcoal MS + 53.7 µM IBA
Jayanthi et al. [10]	Immature male Inflorescence	Callus induction Somatic embryo initiation/maturation/plantlet regeneration Rooting	Y3 + 150 µM 2,4-D; 150 µM Picloram + 3.0 g/L Activated Charcoal Y3 + 18 µM BA; 3.78 µM ABA; 5.78 µM GA
Guedes et al.[40]	Immature female Inflorescence	Callus induction	Y3 + 23 µM IAA; 19.6 µM IBA + 0.5 g/L activated Charcoal
Teixeira et al. [41]	Immature female Inflorescence	Callus induction Somatic embryo initiation/maturation Plantlet regeneration	½ MS + 225–450 µM 2,4-D + 3.0 g/L Activated Charcoal MS + 475 µM 2,4-D + 3.0 g/L Activated Charcoal Y3 + 15 µM NAA; 2 µM ABA ½ MS + 3.0 g/L Activated Charcoal
Jayanthi et al. [44]	Young plantlet	Somatic embryogenesisPlantlet regeneration	Y3 + 40 µM 2,4-D; 10 µM 2,4,5-T; 40 µM NAA; 10 µM TDZ; 10 µM BA + 3.0 g/L Activated Charcoal Y3 + 2 µM BA; 1 µM ABA
Scherwinski-Pereira et al. [45]	Young plantlet	Callus induction Somatic embryo initiation/maturation Plantlet regeneration	MS + 450 µM Picloram + 0.3 g/L Activated Charcoal MS + 0.6 µM NAA; 12.3 µM 2-ip + 0.3 g/L Activated Charcoal ½ MS + 1.0 g/L Activated Charcoal

MS Medium: Murashige and Skoogs medium [49], Y3 Medium [50], N6 Medium [51], ABA: abscisic acid, EC: embryogenic callus, NAA: 1-naphthaleneacetic acid, SE: somatic embryogenesis.

## References

[1] Hormaza P., Fuquen E.M., Romero H.M. (2012). Phenology of the oil palm interspecific hybrid *Elaeis oleifera* × *Elaeis guineensis*. Sci. Agric..

[2] Sumaryono R.I., Saptari R.T., Rahmadi H.Y., Ernayunita (2018). Embryogenic callus initiation from leaf explants of *Elaeis oleifera* x *Elaeis guineensis* (OxG) hybrids. IOP Conf. Series.

[3] Weckx S., Inzé D., Maene L. (2019). Tissue Culture of Oil Palm: Finding the Balance between Mass Propagation and Somaclonal Variation. Front. Plant Sci..

[4] Huth N.I., Banabas M., Nelson P.N., Webb M. (2014). Development of an oil palm cropping systems model: Lessons learned and future directions. Environ. Modell. Softw..

[5] FAOSTAT (2013). Database Food and Agriculture Organization of the United Nations.

[6] Masani M.Y.A., Izawati A.M.D., Rasid O.A., Parveez G.K.A. (2018). Biotechnology of oil palm: Current status of oil palm genetic transformation. Biocat. Agric. Biotechnol..

[7] Corley R.H.V. (2009). How much palm oil do we need?. Environ. Sci. Policy.

[8] Zulkifli Y., Norziha A., Naqiuddin M.H., Fadila A.M., Nor Azwani A.B., Suzana M., Samsul K.R., Ong-Abdullah M., Singh R., Parveez G.K.A. (2017). Designing the oil palm of the future. J. Oil Palm Res..

[9] Mielke T. (2013). Palm Oil the Leader in Global Oils & Fats Supply. http://www.mpoc.org.my/upload/Plenary_Paper-Thomas-Mielke.

[10] Jayanthi M., Susanthi B., Murali Mohan N., Mandal P.K. (2015). In vitro somatic embryogenesis and plantlet regeneration from immature male inflorescence of adult dura and tenera palms of *Elaeis guineensis* (Jacq.). Springer Plus.

[11] Basiron Y. (2007). The Palm-Oil Advantage in Biofuel. New Straits Times.

[12] Green M., Lima W.A.A., de Figueiredo A.F., Atroch A.L., Lopes R., da Cunha R.N.V., Teixeira P.C. (2013). Heat-treatment and germination of oil palm seeds (*Elaeis guineensis* Jacq.). J. Seed Sci..

[13] Kushairi A., Tarmizi A.H., Zamzuri I., Ong-Abdullah M., Samsul Kamal R., Ooi S.E., Rajanaidu N. Production, performance and advances in oil palm tissue culture. Proceedings of the International Society of Oil Palm Breeders (ISOPB) Seminar on Advances in Oil Palm Tissue Culture.

[14] Lee F.C., Ong-Abdullah M., Ooi S.E., Ho C.L., Namasivayam P. (2019). Cloning and characterization of *Somatic Embryogenesis Receptor Kinase I* (*EgSERK I*) and its association with callus initiation in oil palm. In Vitro Cell Dev. Biol. Plant.

[15] Muniran F., Bhore S.J., Shah F.H. (2008). Micropropagation of *Elaies guineensis* Jacq. ‘Dura’: comparison of three basal media for efficient regeneration. Indian J. Exp. Biol..

[16] Tan C.C., Wong G., Soh A.C., Hor T.Y., Chong S.P., Gopal K. Experiences and lessons from oil palm clonal evaluation trials and commercial test plantings. Proceedings of the 2003 PIPOC International Palm Oil Congress, Malaysian Palm Oil Board.

[17] Kushairi A., Singh R., Ong-Abdullah M. (2017). The oil palm industry in Malaysia: Thriving with transformative technologies. J. Oil Palm Res..

[18] Kushairi A., Mohd Din A., Rajanaidu N., Mohd Basri W., Choo Y.M., Chan K.W. (2011). Oil palm breeding and seed production. Further Advances in Oil Palm Research (2000–2010) 1.

[19] Rajanaidu N., Kushairi A., Rafii M., Mohd Din A., Maizura I., Jalani B.S., Basiron Y., Jalani B.S., Chan K.W. (2000). Oil palm breeding and genetic resources. Advances in Oil Palm Research.

[20] Zou J., Zhang Q., Zhu Z., Gao L., Zheng Y., Li D. (2019). Embryogenic callus induction and fatty acid composition analysis of oil palm (*Elaeis guineensis* cv. Tenera). J. Sci..

[21] Padua M.S.S., Santos R.S., Paiva L.V., Steins V.C., Silva L.C. (2017). In vitro rooting of tenera hybrid oil palm (*Elaeis guineensis* Jacq.) plants. Rev. Arvore..

[22] Jones L.H. (1974). Propagation of clonal oil palms by tissue culture. Planter.

[23] Rabéchault H., Martin J.P. (1976). Multiplication végétative du palmier à huile (*Elaeis guineensis* Jacq.) à l’aide de cultures de tissus foliaires. C. R. Acad. Sci. Paris Ser. D.

[24] Staritsky G. (1970). Tissue culture of the oil palm (*Elaeis guineensis* Jacq.) as a tool for vegetative propagation. Euphytica.

[25] Monteiro T.R., Freitas E.O., Nogueira G.F., Scherwinski-Pereira J.E. (2017). Assessing the influence of subcultures and liquidmedium during somatic embryogenesis and plant regeneration in oilpalm (Elaeis guineensis Jacq.). J. Hortic. Sci. Biotechnol..

[26] Feher A., Pasternak T.P., Dudits D. (2003). Transition of somatic plant cells to an embryogenic state. Plant Cell. Tissue Organ Cult..

[27] Hashim A.T., Ishak Z., Rosli S.K., Ong-Abdullah M., Ooi S.-E., Husri M.N., Bakar D.A., Jain S.M., Gupta P. (2018). Oil palm (*Elaeis guineensis* Jacq.) somatic embryogenesis. Step Wise Protocols for Somatic Embryogenesis of Important Woody Plants.

[28] Constantin M., Nchu W.A., Godswill N., Wiendi N.M.A., Wachjar A., Frank N.E.G. (2015). Induction of oil palm (*Elaeis guineensis* Jacq. var. Tenera) callogenesis and somatic embryogenesis from young leaf explants. J. Appl. Biol. Biotechnol..

[29] Eeuwens C.J., Lord S., Donough C.R., Rao V., Vallejo G., Nelson S. (2002). Effects of tissue culture conditions during embryoid multiplication on the incidence of “mantled” flowering in clonally propagated oil palm. Plant Cell. Tiss Org Cult..

[30] Rajesh M.K., Radha E., Karun A., Parthasarathy V.A. (2003). Plant regeneration from embryo-derived callus of oil palm—The effect of exogenous polyamines. Plant Cell. Tiss Org Cult..

[31] Corrêa T.R., Motoike S.Y., de Souza Andrade A.P., Coser S.M., Queiroz V., Granja M.M.C., Caetano D.D.N., Pena C.N.M., Picoli E.A.d.T. (2016). Accelerated in vitro propagation of elite oil palm genotypes (*Elaeis guineensis* Jacq.) by substituting cytokinin with putrescine. Afr. J. Biotechnol..

[32] Ong-Abdullah M., Ordway J.M., Jiang N., Ooi S.E., Kok S.Y., Sarpan N., Azimi N., Hashim A.T., Ishak Z., Rosli S.K. (2015). Loss of Karma transposon methylation underlies the mantled somaclonal variant of oil palm. Nature.

[33] Alwee S.S., Linder V., Schoot J.V.T., Folter S., Angent G.C., Cheach S.C., Smulders M.J.M. (2006). Characterization of oil palm MADS box genes in relation to the mantled flower abnormality. Plant Cell. Tissue Org. Cult..

[34] Gomes H.T., Bartos P.M.C., Balzon T.A., Scherwinski-Pereira J.E. (2016). Regeneration of somatic embryos of oil palm (*Elaeis guineensis*) using temporary immersion bioreactors. Ind. Crops Prod..

[35] Gomes H.T., Bartos P.M.C., Scherwinski-Pereira J.E. (2015). Optimizing rooting and survival of oil palm (*Elaeis guineensis*) plantlets derived from somatic embryos. In Vitro Cell. Dev. Biol..

[36] De Carvalho Silva R., Luis Z.G., Scherwinski-Pereira J.E. (2014). The histo differentiation events involved during the acquisition and development of somatic embryogenesis in oil palm (*Elaeis guineensis* Jacq.). Plant Growth Regul..

[37] Balzon T.A., Luis Z.G., Scherwinski-Pereira J.E. (2013). New approaches to improve the efficiency of somatic embryogenesis in oil palm (*Elaeis guineensis* Jacq.) from mature zygotic embryos. In Vitro Cell. Dev. Biol..

[38] Wan Nur Syuhada W.S., Rasid O.A., Parveez G.K.A. (2016). Evaluation on the effects of culture medium on regeneration of oil palm plantlets from immature embryos (IE). J. Oil Palm Res..

[39] Thuzar M., Vanavichit A., Tragoonrung S., Jantasuriyarat C. (2011). Efficient and rapid plant regeneration of oil palm zygotic embryos cv. “Tenera” through somatic embryogenesis. Acta Physiol. Plant..

[40] Guedes S., Da R., da Silva T.L., Luis Z.G., Scherwinski-Pereira J.E. (2011). Initial requirements for embryogenic calluses initiation in thin cell layers explants from immature female oil palm inflorescences. Afr. J. Biotechnol..

[41] Teixeira J.B., Söndahl M.R., Kirby E.G. (1994). Somatic embryogenesis from immature inflorescences of oil palm. Plant Cell Rep..

[42] Gomes H.T., Bartos P.M.C., Scherwinski-Pereira J.E. (2017). Dynamics of morphological and anatomical changes in leaf tissues of an interspecific hybrid of oil palm during acquisition and development of somatic embryogenesis. Plant Cell. Tissue Organ Cult..

[43] De Touchet B., Duval Y., Pannetier C. (1991). Plant regeneration from embryogenic suspension cultures of oil palm (*Elaeis guineensis* Jacq.). Plant Cell Rep..

[44] Jayanthi M., Mohan N.M., Mandal P.K. (2011). Direct somatic embryogenesis and plantlet regeneration in oil palm. J. Plant Biochem. Biotechnol..

[45] Scherwinski-Pereira J.E., da Guedes R.S., Fermino P.C.P., Silva T.L., Costa F.H.S. (2010). Somatic embryogenesis and plant regeneration in oil palm using the thin cell layer technique. In Vitro Cell. Dev. Biol..

[46] Romyanon K., Mosaleeyanon K., Kirdmanee C. (2015). Direct-shoot organogenesis as an alternative protocol for in vitro regeneration of oil palm (*Elaeis guineensis* Jacq.). Sci. Hortic-Amsterdam.

[47] Ho W.K., Ooi S.E., Mayes S., Namasivayam P., Ong-Abdullah M., Chin C.F. (2013). Methylation levels of a novel genetic element, *EgNB3* as a candidate biomarker associated with the embryogenic competency of oil palm. Tree Genet. Genomes..

[48] Mariani T.S., Sasmitamiharja D., Mienanti D., Latif S., Ginting G., Miyake H. (2014). Somatic Embryogenesis of Oil Palm (*Elaeis guineensis* Jacq.) for Synthetic Seed Production. As. J. Appl. Sci..

[49] Murashige T., Skoog F. (1962). A revised medium for rapid growth and bio assays with tobacco tissue cultures. Physiol. Plant..

[50] Eeuwens C.J. (1978). Effects of organic nutrients and hormones on growth and development of tissue explants from coconut (*Cocos nucifera*) and Date (*Phoenix dactylifera*) palms cultured in vitro. Physiol. Plant..

[51] Chu C.C., Wang C.C., Sun C.S., Hsu C., Yin K.C., Chu C.Y., Bi F.Y. (1975). Establishment of an efficient medium for another culture of rice through comparative experiments on nitrogen sources. Sci. Sin..

[52] Bakoumé C., Ahuja M., Jain S. (2018). Genetic Diversity, Erosion, and Conservation in Oil Palm (*Elaeis guineensis* Jacq.). Genetic Diversity and Erosion in Plants.

[53] Teixeira da Silva J.A., Engelmann F. (2017). Cryopreservation of oil palm (*Elaeis guineensis* Jacq.). Cryobiology.

[54] Engelmann F., Duval J.Y. (1985). Dereuddre Survival and proliferation of oil palm (*Elaeis guineensis* Jacq.) somatic embryos after freezing in liquid nitrogen. C. R. Acad. Sci. Paris.

[55] Parveez G.K.A., Rasid O.A., Masani M.Y.A., Sambanthamurthi R. (2015). Biotechnology of oil palm: Strategies towards manipulation of lipid content and composition. Plant Cell Rep..

[56] Izawati A.M.D., Masani M.Y.A., Ismanizan I., Parveez G.K.A. (2015). Evaluation on the effectiveness of 2-deoxyglucose-6-phosphate phosphatase (*DOG^R^1*) gene as a selectable marker for oil palm (*Elaeis guineensis* Jacq.) embryogenic calli transformation mediated by *Agrobacterium tumefaciens*. Front. Plant Sci..

[57] Hashim A.T., Ishak Z., Ooi S.E., Rosli S.K., Chan P.L., Rohani O., Ong-Abdullah M., Wahid M.B., Choo Y.M., Chan K.W. (2011). Forging ahead with clones. Further Advances in Oil Palm Research.

[58] Parveez G.K.A., Jain S.N., Minocha S.C. (2000). Production of transgenic oil palm (*Elaeis guineensis* Jacq.) using biolistic techniques. Molecular Biology of Woody Plants 2.

[59] Sanford J.C., Klein T.M., Wolf E.D., Allen N. (1987). Delivery of substances into cells and tissues using a particle bombardment process. J. Part. Sci. Tech..

[60] Christou P., McCabe D.E., Swain W.F. (1988). Stable transformation of soybean callus by DNA coated particles. Plant Physiol..

[61] Klein T.M., Harper E.C., Svab Z., Sanford L.C., Fromm M.E., Maliga P. (1988). Stable genetic transformation of intact *Nicotiana* cells by the particle bombardment process. Proc. Natl. Acad. Sci. USA.

[62] Finer J.J., Finer K.R., Ponappa T., Hammond J., McGarvey P., Yusibov V. (2000). Particle Bombardment Mediated Transformation. Current Topics Microbiology and Immunology.

[63] Parveez G.K.A., Chowdhury M.K.U., Saleh N.M. (1998). Biological parameters affecting transient GUS gene expression in oil palm (*Elaeis guineensis* Jacq.) embryogenic calli via microprojectile bombardment. Ind. Crops Prod..

[64] Parveez G.K.A., Chowdhury M.K.U., Saleh N.M. (1997). Physical parameters affecting transient GUS gene expression in oil palm (*Elaeis guineensis* Jacq.) using the biolistic device. Ind. Crops Prod..

[65] Chowdhury M.K.U., Parveez G.K.A., Saleh N.M. (1997). Evaluation of five promoters for use in transformation of oil palm (*Elaeis guineensis* Jacq.). Plant Cell Rep..

[66] Parveez G.K.A., Chowdhury M.K.U., Saleh N.M. (1996). Determination of minimal inhibitory concentration of selection agents for oil palm (*Elaies guineesis* Jacq.) transformation. As. Pac. J. Mol. Biol. Biotechnol..

[67] Parveez G.K.A., Christou P. (1998). Biolistic-mediated DNA delivery and isolation of transgenic oil palm (*Elaeis guineensis* Jacq.) embryogenic callus cultures. J. Oil Palm Res..

[68] Nurfahisza A.R., Rafiqah M.A., Masani M.Y.A., Hanin A.N., Rasid O.A., Parveez G.K.A., Ismail I. (2014). Molecular analysis of transgenic oil palm to detect the presence of transgenes. J. Oil Palm Res..

[69] Nurfahisza A.R., Rafiqah M.A., Parveez G.K.A., Rasid O.A. (2016). Comparison of the effectiveness of Basta, bialaphos and glufosinate ammonium for selecting transformed oil palm tissues. J. Oil Palm Res..

[70] Bahariah B., Parveez G.K.A., Khalid N. (2012). Determination of optimal concentration of mannose as a selection agent for selecting transformed oil palm cells using the phosphomannose isomerase (pmi) gene as the positive selectable marker. J. Oil Palm Res..

[71] Bahariah B., Parveez G.K.A., Masani M.Y.A., Masura S.S., Khalid N., Othman R.Y. (2013). Biolistic transformation of oil palm using the phosphomannose isomerase (*pmi*) gene as a positive selectable marker. Biocatal. Agric. Biotechnol..

[72] Masli D.I.A., Parveez G.K.A., Ismail I. (2012). Optimisation of 2-deoxyglucose concentration for identifying the sensitivity level for oil palm embryogenic calli. J. Oil Palm Res..

[73] Parveez G.K.A., Bahariah B., Ayub N.H., Masani M.Y.A., Rasid O.A., Tarmizi A.H., Ishak Z. (2015). Production of polyhydroxybutyrate in oil palm (*Elaeis guineensis* Jacq.) mediated by microprojectile bombardment of PHB biosynthesis genes into embryogenic calli. Front. Plant Sci..

[74] Tzfira T., Citovsky V. (2006). *Agrobacterium*-mediated genetic transformation of plants: Biology and biotechnology. Curr. Opin. Biotech..

[75] Raineri D.M., Bottino P., Gordon M.P., Nester E.W. (1990). *Agrobacterium tumefaciens* mediated transformation of rice (*Oryza sativa* L.). Bio/Technology.

[76] Ahmed R.I., Ding A., Xie M., Kong Y. (2018). Progress in Optimization of *Agrobacterium*-Mediated Transformation in Sorghum (*Sorghum bicolor*). Int. J. Mol. Sci..

[77] Koetle M.J., Finnie J.F., Balázs E., Van Staden J. (2015). A review on factors affecting the *Agrobacterium*-mediated genetic transformation in ornamental monocotyledonous geophytes. S. Afr. J. Bot..

[78] Hiei Y., Ishida Y., Komari T. (2014). Progress of cereal transformation technology mediated by *Agrobacterium tumefaciens*. Front. Plant Sci..

[79] Masli D.I.A., Parveez G.K.A., Yunus A.M.M. (2009). Transformation of oil palm using *Agrobacterium tumefaciens*. J. Oil Palm Res..

[80] Izawati A.M.D., Parveez G.K.A., Masani M.Y.A. (2012). Transformation of oil palm using *Agrobacterium tumefaciens*. Methods Mol. Biol..

[81] Yenchon S., Te-chato S. (2012). Effect of bacteria density, inoculation and cocultivation period on *Agrobacterium*-mediated transformation of oil palm embryogenic callus. J. Agric. Technol..

[82] Promochan T., Te-Chato S. (2013). Strains of *Agrobacterium* affecting gene transformation through embryogenic cell suspension of hybrid tenera oil palm. J. Agric. Technol..

[83] Parveez G.K.A. (1998). Optimization of Parameters Involved in the Transformation of Oil Palm Using the Biolistics Method. Ph.D. Thesis.

[84] Masani M.Y.A., Noll G., Parveez G.K.A., Sambanthamurthi R., Prüfer D. (2013). Regeneration of viable oil palm plants from protoplasts by optimizing media components, growth regulators and cultivation procedures. Plant Sci..

[85] Masani M.Y.A., Noll G., Parveez G.K.A., Sambanthamurthi R., Pruefer D. (2014). Efficient transformation of oil palm protoplasts by PEG-mediated transfection and DNA microinjection. PLoS ONE.

[86] Chen K., Wang Y., Zhang R., Zhang H., Gao C. (2019). CRISPR/Cas Genome Editing and Precision Plant Breeding in Agriculture. Ann. Rev. Plant Bio..

[87] Haque E., Taniguchi H., Hassan M.M., Bhowmik P., Karim M.R., Śmiech M., Zhao K., Rahman M., Islam T. (2018). Application of CRISPR/Cas9 Genome Editing Technology for the Improvement of Crops Cultivated in Tropical Climates: Recent Progress, Prospects, and Challenges. Front. Plant Sci..

[88] Sattar M.N., Iqbal Z., Tahir M.N., Shahid M.S., Khurshid M., Al-Khateeb A.A., Al-Khateeb S.A. (2017). CRISPR/Cas9: A Practical Approach in Date Palm Genome Editing. Front. Plant Sci..

[89] Abdullah S.N.A., Azzeme A.M., Ebrahimi M., Ariff E.A.K.E., Hanifiah F.H.A., Abdullah S., Chai-Ling H., Wagstaff C. (2017). Transcription Factors Associated with Abiotic Stress and Fruit Development in Oil Palm. Crop Improvement.

[90] Arshad C.M., Armanto M.E., Zain A.M. (2012). Evaluation of climate suitability for oil palm (*Elaeis guineensis* Jacq.) cultivation. J. Environ. Sci. Eng..

[91] Singh R., Ong-Abdullah M., Low E.T., Manaf M.A., Rosli R., Nookiah R., Ooi L.C., Ooi S.E., Chan K.L., Halim M.A. (2013). Oil palm genome sequence reveals divergence of interfertile species in Old and New worlds. Nature.

[92] Xiao Y., Zhou L., Lei X., Cao H., Wang Y., Dou Y., Tang W., Xia W. (2017). Genome-wide identification of WRKY genes and their expression profiles under different abiotic stresses in *Elaeis guineensis*. PLoS ONE.

